# Implementation of a comprehensive sickle cell center leads to improved access to care and decreased acute care use

**DOI:** 10.1186/s12913-026-15206-6

**Published:** 2026-07-24

**Authors:** Julie Kanter, Allison Plaxco, Najibah Galadanci, Gerhard Helleman, Rachel Klein, Jeffrey Lebensburger

**Affiliations:** 1 Lifespan Comprehensive Sickle Cell Center, University of Alabama Birmingham, Birmingham, AL USA; 2 Department of Medicine, University of Alabama Birmingham, Birmingham, AL USA; 3 School of Public Health, University of Alabama Birmingham, Birmingham, AL USA; 4 Department of Pediatrics, University of Alabama Birmingham, Birmingham, AL USA; 5 Division of Hematology and Oncology, Department of Medicine, University of Alabama at Birmingham, Birmingham, AL USA

**Keywords:** Sickle cell disease, Comprehensive sickle cell clinic, Access, Acute care

## Abstract

**Background:**

Sickle cell disease (SCD) is a lifespan condition associated with substantial morbidity, premature mortality, and high healthcare utilization, particularly due to acute pain crises and other severe complications. Evidence-based guidelines emphasize the importance of comprehensive, multidisciplinary care for optimal outcomes; however, implementation of such models for adults with SCD remains inconsistent, contributing to fragmented care and reliance on emergency services. The primary aim of the study is to evaluate the implementation and impact of a National Alliance of Sickle Cell Centers (NASCC) aligned comprehensive adult SCD center on outpatient engagement, infusion therapy utilization, and acute care use.

**Methods:**

The University of Alabama at Birmingham implemented a multidisciplinary adult SCD center beginning in 2019. We conducted a retrospective, population-based evaluation comparing care engagement and utilization before (January 2017–December 2018) and after (January 2022–December 2024) center implementation, excluding the COVID-19 period. Electronic medical record data were used to identify adults with SCD and capture outpatient clinic visits, infusion therapy unit use, emergency department (ED) visits, and hospitalizations. Nonparametric statistical tests were used to compare outcomes across periods.

**Results:**

The adult SCD clinic population increased more than three-fold, reaching 810 patients by 2024, alongside substantial expansion of clinical space, staffing, and infusion capacity. By 2024, nearly 90% of patients with SCD were seen annually in the outpatient clinic. Infusion therapy visits increased fifteen-fold, providing an alternative to ED-based pain management. Mean acute care visits per person per year decreased significantly from 2.11 to 1.26, while mean routine outpatient visits per person per year increased from 2.65 to 5.32 (*p* < 0.001). Reductions in ED visits and hospitalizations were observed despite a marked increase in the total population served.

**Conclusions:**

Implementation of a NASCC-aligned comprehensive adult SCD center was associated with improved outpatient engagement, expanded access to preventive and infusion-based care, and significant reductions in acute care utilization. These findings provide real-world evidence that comprehensive, multidisciplinary SCD centers are an effective structural strategy to improve care delivery and outcomes for adults living with SCD and support broader adoption of this model to advance equity and value-based care.

**Clinical trial number:**

Not applicable.

## Introduction

Sickle cell disease (SCD) is a lifespan disease that disproportionately impacts historically marginalized populations, contributing to substantial morbidity, premature mortality, and high healthcare utilization [1, 2]. Acute pain crisis (APC) is the most common reason for affected individuals to present to an emergency department (ED). However, in addition to pain, there are several other acute complications of SCD including acute chest syndrome, stroke, severe anemia, and infections that are superimposed on chronic, cumulative organ damage throughout the lifespan. These complex medical co-morbidities, combined with associated psychosocial stressors require coordinated, longitudinal screening and multi-disciplinary management for optimal outcomes [3–5]. 

Evidence-based guidelines emphasize that high-quality SCD care depends on reliable access to preventive and disease-modifying strategies to ensure longitudinal monitoring for end-organ complications and structured approaches to pain management [3, 4, 6, 7]. With such a complex disease requiring lifespan multidisciplinary care, the translation and implementation of guideline recommendations into consistent real-world practice remains challenging.

Compared to other chronic, inherited conditions such as cystic fibrosis and hemophilia, SCD is underfunded and associated clinics are often understaffed and minimally supported [8–10]. In pediatric departments, staffing is often provided in combination with oncology care to ensure affected children can access necessary supportive services including social work, psychology, and educational assistance. Unfortunately, adult-focused SCD centers are often more resource constrained and geographically limited. Thus, the care received by adults with SCD may be fragmented across emergency, inpatient, outpatient, and community settings.

To address the national barriers that impact patient outcomes, the National Alliance of Sickle Cell Centers (NASCC) was established to define the necessary components of SCD centers in order to enhance the delivery of high-quality comprehensive care by setting standards and promoting their adoption, with a focus on improving access and outcomes [11]. Using this model, SCD centers are intentionally designed to prevent care fragmentation through the delivery of integrated, longitudinal, team-based care that combines specialty expertise with standardized protocols, care coordination, and wraparound psychosocial support [11, 12]. Recent NASCC publications on the definition of a SCD center and consensus recommendations for lifespan annual screening assessments as well as the use of a structured transition documentation form further highlight these efforts to improve care [12]. These NASCC consensus recommendations emphasize the importance of having a multi-disciplinary team that includes a physician who is a SCD specialist, educated nurses and advance practice providers, mental health, care coordination and community-based organizations.

## Setting

The University of Alabama at Birmingham developed and implemented a comprehensive SCD center starting in February of 2019. Prior to this time, the institution had a long history of providing care for individuals with SCD using a traditional consultative clinic model but did not have a dedicated, multi-disciplinary program. In this setting, outpatient clinic occurred one day/week for routine wellness and did not include wrap-around services with only minimal infusion capacity beyond the need for transfusion therapy. Under the combined leadership of the Departments of Medicine and Pediatrics at UAB and a supportive donation from the local community-based organization (CBO), the decision was made to develop a multi-disciplinary SCD center for adults aligned with current NASCC recommendations lead by a SCD specialist. Notably, the majority of the funding was provided by UAB with the recognition that there is a large population of people with SCD in Alabama and that improved preventative care could decrease acute care utilization (alleviating the crowded emergency departments and providing more hospital bed space for other conditions). Initially, clinic and ITU space (as well as staff) were limited until proof of concept were established. The donation from the CBO was used to fund one of the social workers for the adult program but this role (and the additional social workers provided later) were later provided through standard transition care funding (i.e. hospital funding).

**Fig. 1:**
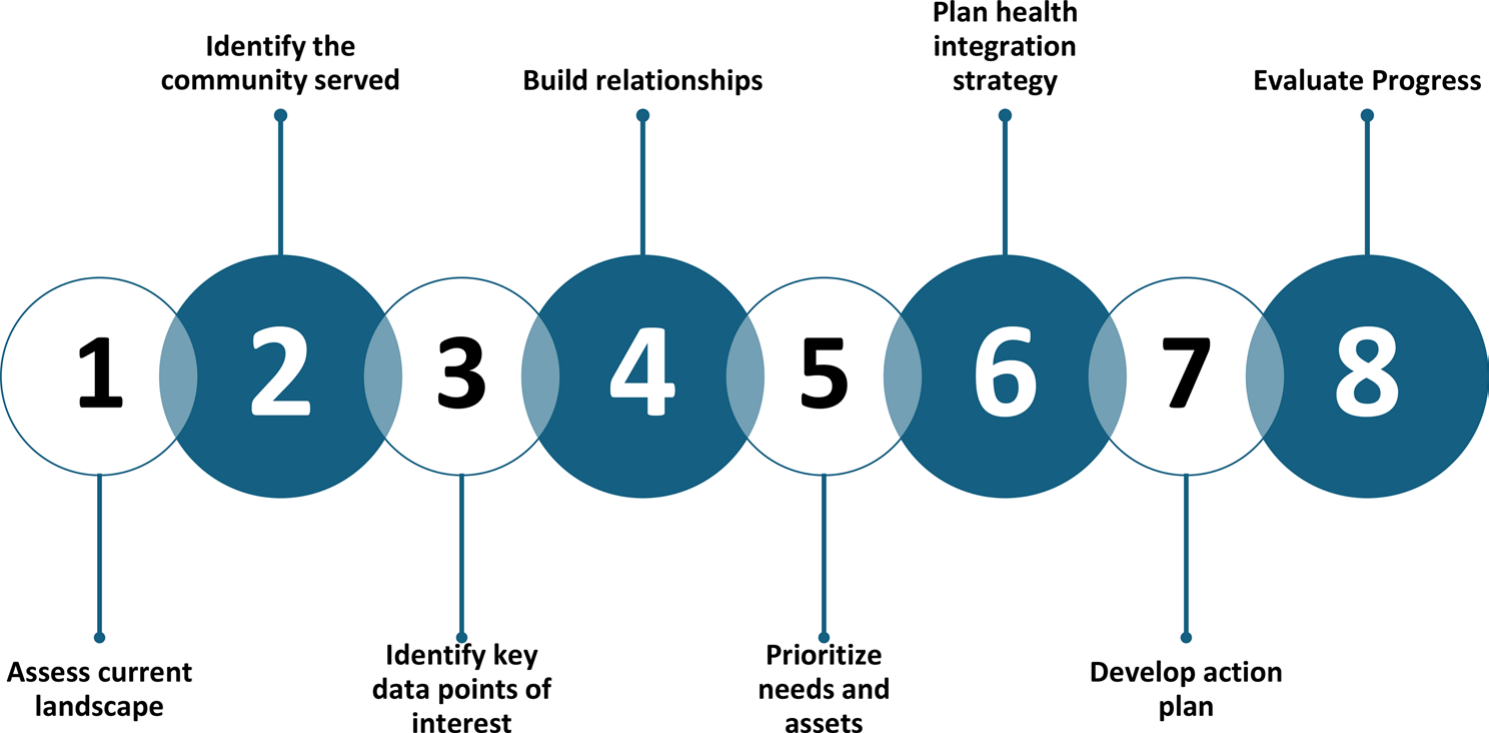
Comprehensive needs assessment

**Fig. 2:**
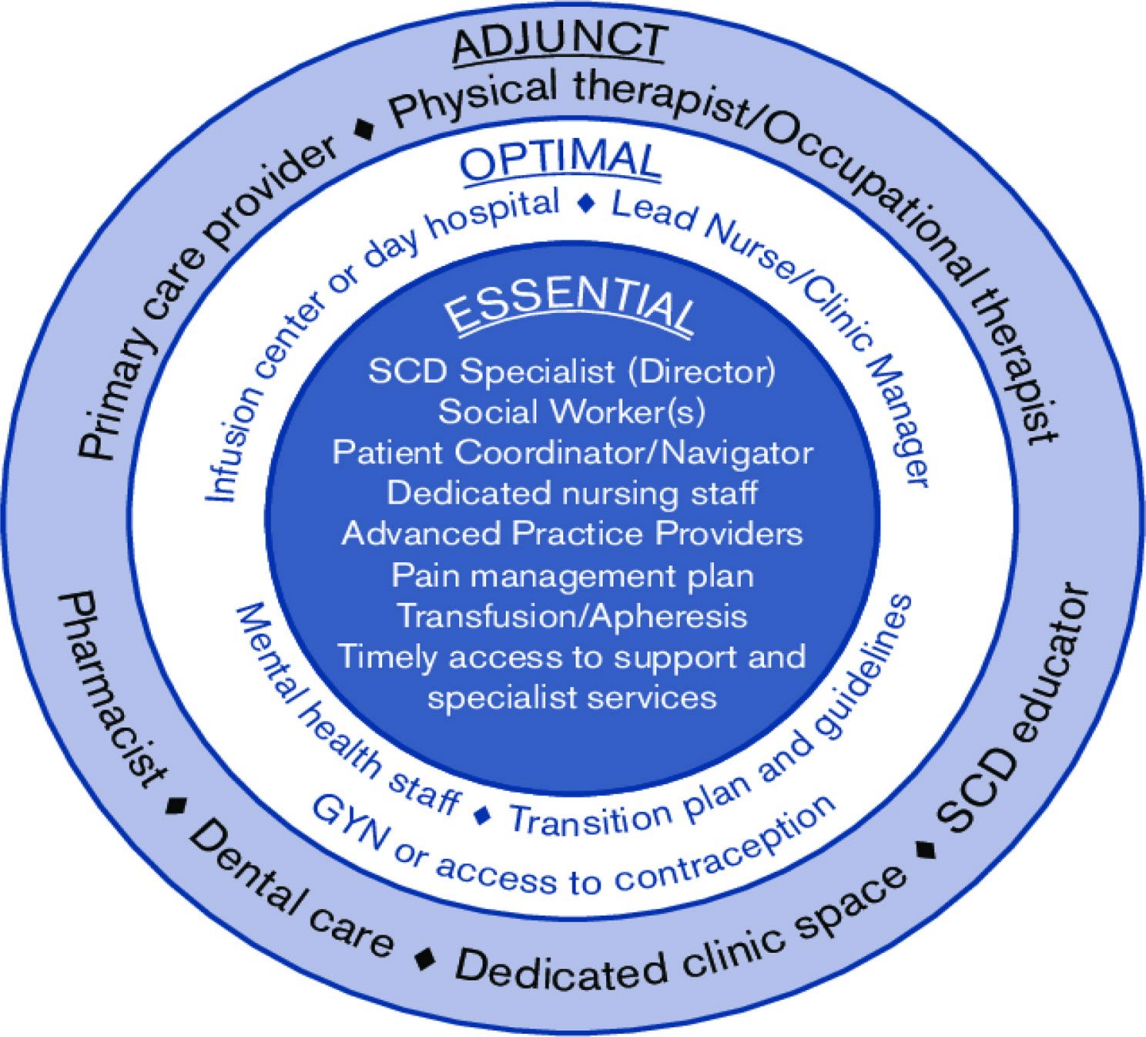
The components of a SCD comprehensive center

A systematic approach to monitor healthcare utilization outcomes was initiated at the onset of the SCD center implementation to ensure the return on investment could be documented to encourage the hospital system to sustain the program. We first performed a comprehensive needs assessment to understand the size and location of the patient population, health utilization patterns, available personnel and space, and to assess the availability of SCD specific education, knowledge, policies and practices within the hospital. The steps undertaken are shown in Fig. 1 and key findings described below. The areas evaluated were specifically aligned with the requirements of a NASCC-defined comprehensive center (Fig. 2).

### Population data

There are two separate (overlapping) patient populations with SCD that will be discussed in detail below. (1) UAB SCD Patient Population: This cohort will show population-level results from everyone who obtained care at UAB for SCD during the study period. This cohort of patients is defined by applying the sickle cell data collection (SCDC) definitions (three ICD-9/10 codes for SCD in a five-year period) [13]. The population cohort will serve as the broader denominator for health care utilization measures such as ED visits, hospitalizations, and overall utilization. (2) UAB SCD Center Cohort: The population of patients with SCD who engaged in the SCD Center will be included in this cohort. Notably, these same persons are also in first cohort (SCD Population) but this is a more defined patient group who were seen for routine, non-acute care in the SCD clinic. The Center Cohort will help demonstrate the effect of comprehensive, multi-disciplinary care.

When we identified the SCD population definition (above), we identified approximately 1300 adults with SCD within the UAB system (outpatient, emergency department and hospital) between 2016 and 2018. However, only 20% of these adults with SCD (*n* = 273) were followed by the UAB outpatient hematology clinic (Center Cohort). There were several reasons for this disconnect. (1) The lack of a formal referral process for persons seen in the acute care facilities to the SCD clinic for outpatient care. (2) Many established adults with SCD travelled from counties throughout Alabama (outside of Birmingham) including from Mississippi and were not seen regularly. (3) Additional adults with SCD living outside of Birmingham were not necessarily referred to the SCD clinic if they were followed by a community oncologist. These original 1300 persons were the target of the initial needs assessment performed to improve SCD Care at UAB.

### Clinic and infusion space/staffing

The initial needs assessment highlighted the need for additional space both for routine clinical care and infusion for pain management. The clinic rooms initially used for SCD care were located in a shared multi-specialty clinic with other specialties and only two clinic rooms were available on Fridays. The UAB hospital infusion capacity included only three infusion areas one of which was prioritized for persons with cancer (needing chemotherapy) and the others were shared with the rest of the hospital. Thus, there was very limited capacity for treating acute pain. Staffing was also limited and there was only a single assigned nurse, a care coordinator and one social worker for the SCD clinic. We recognized a clear need for additional space to ensure patients with SCD could be seen daily, a dedicated infusion unit or day hospital for acute pain management, and additional staff including nurses and medical assistants to enhance the program.

### Transition program

A second barrier identified in our needs assessment was the lack of a formalized transition process from the Children’s of Alabama to UAB. The transition plan was limited to transfer of care by providing the names of transition age patients to an adult social worker who helped facilitate their initial visit at UAB. We identified more than 75 patients over 18 years of age awaiting transition to adult care at this time; however, a routine assessment of a successful transition was not performed. Thus, the implementation of the SCD center also required the initiation of a formal transition program to improve the transfer of care from the pediatric SCD program to the adult center [14]. 

### Transfusion and apheresis

Third, we identified that while the blood bank and apheresis team were very knowledgeable in SCD transfusion care, there was limited care coordination with the SCD team. Blood for transfusion was C/E/Kell matched and patients with SCD underwent a phenotype upon entry into the UAB SCD system of care. Those patients with SCD who had been identified with a history of stroke in pediatrics were started on chronic transfusion therapy or apheresis. However, there were a few (< 5%) started on chronic transfusion by the adult providers. Finally, the apheresis team used a generic SCD treatment plan as opposed to individualized transfusion plans and iron overload was assessed with ferritin measurements, but liver MRI had not been adopted. The findings allows us to identify a need for improved care coordination for patients needing chronic red cell transfusion and an updated approach to iron overload assessment.

### Horizontal integration of services

As of 2019, there were very few opportunities for a horizontal approach to patient care across the disciplines within the larger hospital infrastructure. Horizontal integration of services requires that individual patients receive the same care plan throughout a larger medical establishment [15]. Like the use of antimicrobial stewardship for the prevention of antibiotic-resistance, a horizonal intervention in SCD includes a clear, system-wide plan for management of the population. Overall, horizontal interventions are considered more cost-effective and have a broader impact. Further, the use of horizontal care integration is a method for improving care equity [16]. In the setting of SCD, it is necessary to ensure a horizontal strategy for pain management as well as for other areas such as peri-operative and obstetric care.

Acute pain crisis is the most frequent cause of emergency department (ED) and inpatient care for people with SCD [17, 18]. Historically, people with SCD have experienced systemic inequities in hospital care, including suboptimal pain management and pervasive stigma [19, 20]. To reduce inequities in pain management, horizontally integrated, team-based strategies across care settings are needed to ensure consistent, evidence-based treatment in both outpatient and inpatient environments. Despite guidelines in SCD recommending the use of individualized care plans (ICP) [7] for affected persons, the clinic at UAB had not initiated this intervention. Further, there had not been any discussions around the need to harmonize care between the outpatient clinic, emergency department (ED) or inpatient unit.

Similarly, there was a recognized need for specialized peri-operative and obstetric care management in SCD but there were no strategies in place to ensure this occurred. When the adults with SCD were admitted to the hospital, they were cared for a primary service team (hospitalists, obgyn, surgery, etc.) and the consultation of the hematology team was reserved for the need for exchange transfusions. There were attempts made to prepare patients with SCD for surgeries if those individuals were being seen routinely in the clinic, but larger efforts had not been undertaken. Based on these findings, we identified a need to also systematically assess the implementation needs of emergency medicine, hospital medicine, anesthesia/peri-operative, and obstetrics–gynecology teams to address identified gaps in pain management and care delivery.

### Relationship with community based organizations

Alabama is fortunate to have multiple community-based organizations (CBO) who serve the SCD community within the state Historically, discussions were limited to the local CBO in Birmingham and focused on persons needing financial, transportation, or educational assistance. There were no advocacy or educational activities coordinated throughout the state and the CBO had a limited presence in clinic. There was also limited engagement between the hematology physician in the clinic and the CBO personnel.

## Intervention

The interventions were staged throughout the first six months of 2019 and are detailed in table two. The first intervention required an expansion of the dedicated space for SCD center. With a large patient population yet limited space available in the current hematology-oncology clinic space, it was clear that additional outpatient clinical space was required for the new SCD center. To address the very limited infusion and transfusion capacity, a separate infusion therapy unit (day hospital) was also a recognized requirement. Finally, office space was added for support staff as well as the need for more advanced practice providers, social workers, and dedicated nursing staff for both the clinic and infusion unit.

In addition to adapting team composition and clinical space, we intentionally implemented system-wide education and awareness initiatives to support consistent integration of SCD–specific practice changes across care settings. As above, the success of horizontal integration of care practices requires the collaboration of healthcare providers from different departments to ensure all providers are working towards the same goals to decrease error and streamline care practices for specific patient populations. Importantly, successful implementation of horizontally integrated care practices requires permission from hospital leadership as well as early and sustained engagement and support during pre-implementation planning, education, and practice change. At the University of Alabama at Birmingham, administrative engagement began prior to 2019 with the recruitment of a dedicated sickle cell disease specialist and included structured discussions with departmental and divisional leaders, as well as hospital and School of Medicine leadership. Pre-implementation meetings with multiple services detailed in Table 1 were initiated prior to implementation to ensure stakeholders understood and were in alignment throughout the larger institution. At the request of multiple services, SCD specific order sets for use within the electronic medical record (EMR) were created collaboratively to promote engagement with individual providers. The use of individualized care plans (ICPs) was also detailed throughout the hospital to explain their use and improve implementation processes.

The development of the transition program was a joint effort between the pediatric and adult SCD teams to optimize communication and patient transfer. Concurrently, UAB pediatric and adult teams also joined the ST3P-up study to enhance transition education and peer-mentoring [21]. The design included a monthly transition clinic held within the pediatric clinic attended by both the pediatric and adult SCD teams, as pediatric and adult transition coordinators, and social work. As part of the ST3P-UP study, the pediatric transition coordinator reviewed the got-transition elements with patients prior to these appointments and assessed transition readiness accordingly. Ongoing monthly meetings were established to enhance communication and to improve practice harmonization between the pediatric and adult programs teams (pain management, prescription policies, transfusion approaches, etc.) to facilitate and soften the transition of care whenever possible. Data regarding the pre-transition program outcomes is available in a separate publication, and a post-transition publication will be forthcoming [22]. 

To improve continuity of care with the apheresis and blood banking program, a dedicated, monthly transfusion meeting was initiated to enhance multi-disciplinary communication between the blood bank, apheresis, and SCD teams especially as more patients were referred for transfusion services. Simultaneously, the use of red cell genotyping was initiated to enhance the RBC matching starting with patients on chronic red cell therapy (CRCT). Discussions with apheresis and clinic nursing lead to the use of more individualized transfusion goals and targets including the judicious use of red cell depletion with apheresis. Patient histories were reviewed and anyone with a history of stroke or CNS vasculopathy were offered CRCT. Meetings with radiology allowed for the development and use of a liver MRI protocol to improve the assessment and treatment of iron overload.

Simultaneous with the intrahospital education, additional efforts were made with the community-based organizations (CBO) across the state. Instead of limiting the engagement to the local CBO, intentional efforts were undertaken to engage all the AL CBOs since patients with SCD come from many areas within the state. Thus, it was very important to meet and establish rapport with all the stakeholders serving the community. Early discussions with the CBOs also enabled enhanced community engagement and buy-in to the changes at the institution. We also established a referral pathway from each CBO to the UAB SCD center for adults who were unaffiliated from care (in need of a SCD specialist). Further, a community advisory board was implemented involving representation from multiple counties.

**Table 1:** Service lines targeted for SCD education at the time of program implementation

Target area	Goal
Emergency Department	Education on use of ICP (individualized care plans) Development of standardized order sets Discussion around avoidance of opioid prescribing from the ED Follow-up practices Practice harmonization
Hospitalist Group	Development of standardized order sets Education on use of ICP Communication planning for hospital admissions and follow-up Practice harmonization Development of an electronic consult template
Transfusion Medicine	Ensure phenotype matching for people with SCD Improve blood utilization Initiate and optimize process for allogeneic hematopoietic stem cell transplant and gene therapies
Stem Cell Transplant Team	Develop referral practices Discuss treatment practices and develop order sets Identify optimal communication methods between teams Structure follow-up plans
Pre-operative Team	Education on SCD operative management Identification of persons unaffiliated with SCD care Development of preoperative pathways
Orthopedic Surgery team	Education on SCD operative management Optimization of patients for joint replacement procedures Care pathway development
Community Based Organization	Improved referral pathways Education about novel sickle cell disease therapies Discussions about ensuring access to care for patients throughout the state

## Methods

To assess the implementation of the multi-disciplinary SCD center at both the patient, SCD Center and UAB population level, data collection procedures were initiated early in 2019 to capture the use of the infusion therapy unit, clinic visits, unique patients followed by the program as well as the emergency department and hospital utilization rates.

Data collected on acute care utilization and clinic attendance are presented from two distinct time frames: January 2017 through December 2018, representing the period prior to the establishment of the SCD center, and January 2022 through December 2024, reflecting outcomes several years after the center’s implementation. Notably, the data set purposefully omits the years affected by the COVID-19 pandemic to avoid potential confounding and ensure the accuracy and reliability of the findings.

Data were extracted from the electronic medical records for individuals >/=18 years of age who meet the SCDC case definition consistent with the pre-implementation data to identify persons with SCD. To be included in the SCD Center-patient specific analysis, we identified those individuals seen in the sickle cell clinic using location codes at least once outpatient during the study years. A SCD Center database was maintained to assess annual follow-up as discussed below.

The list of clinic and hospital visits were deduplicated by patient and year to identify the number of unique clinic patients over the study period. Acute care visits were identified as those to the ED department or as those that resulted in a hospital admission within the study period. Acute visits were grouped into three categories including ED treat and discharge (EDTD) visits, ED visits that resulted in inpatient admission, and inpatient admissions that did not originate in the ED. These acute visits were tabulated to identify the number of unique people with SCD seeking acute care annually and to calculate the number of acute visits per person per year. Infusion therapy visits and unique infusion therapy patients in the same timeframe were also identified, but these were not centralized prior to the implementation of the comprehensive sickle cell center. To optimally evaluate true acute care use, we excluded all visits for patients undergoing allogeneic transplant or gene therapy. Further, we excluded patients who initiated care at an outside hospital and were transferred to UAB to optimally evaluate the care provided by the SCD center as well as those with prolonged hospital stays > 30 days.

Descriptive statistics are reported including frequencies by category, averages, and percentages. Nonparametric tests were conducted to assess the significance of the difference seen before and after the implementation of the comprehensive SCD center with respect to both acute and one-time outpatient visits per person per year among those who sought care (acute, outpatient, or both) at UAB in either period. Because it is possible for patients to be distinct to one time period or to be represented in both, a violation of the classic independence assumption that comes with many statistical tests for the differences between two groups, analysis was conducted in two segments. One segment contained only those individuals who were unique to a single time period or the other, and the other segment contained only those individuals who were present in both. The Wilcoxon rank sum test was used to assess the former, and either the Wilcoxon signed rank test or the sign test was used to assess the latter, depending on the acceptability of the symmetry assumption for the Wilcoxon signed rank test. All analysis was conducted using R version 4.5.1.

## Results

### SCD clinic population, staffing, and space

The patient population treated by the Adult SCD program (SCD Center Cohort) increased substantially from 2019 to 2024 due to efforts to improve transition of care from the pediatric program, identification and recruitment of persons with SCD unaffiliated from care, and enhanced efforts to ensure patients came in for preventative care. Any person with SCD is welcome to the adult-SCD clinic regardless of their distance from clinic. The only exception is for people with third-party payers not accepted by the institution who cannot or does not want to pay out of pocket (although in this scenario, all efforts are made to work with the individual to change insurance to an accepted option). People without insurance (with SCD) are also seen in the UAB SCD adult clinic though payment is required on a sliding scale. Further, our social work team works closely with people in this situation to identify potential payer options. The UAB SCD program has always served the same geographic area (most of Alabama and the eastern border of Mississippi as well as the northern panhandle of Florida) but more intentional effort was made to inform persons of the program through the CBO. Prior to the implementation of this NASCC-recognized program, persons with SCD could be seen for preventative/routine care weekly and could be seen for acute or urgent issues if there was time or space during the week (if not-they were directed to the emergence department). Starting in 2019, the clinic was open for both preventative and acute care from Monday-Friday from 8am to 5pm.

From 2019 to 2023, the SCD Center Cohort increased from 273 persons to 621 persons exceeding the initial designated clinical space. In September of 2022, a planned evaluation was undertaken with a presentation to the UAB administration to demonstrate areas of progress as well as areas of ongoing need. Most notably, there was insufficient space and staff to care for the increased population. A new business proposal was drafted highlighting areas of care improvements as well as ongoing challenges. The hospital and clinic administration agreed to invest in new space with an enlarged ITU and appropriate staffing noting the improvements. In 2023, the clinic was moved to a new space to accommodate the increased population and increase infusion capacity. The original SCD clinic included 4 exam rooms and 6 infusion chairs with registered nurse (RN) staffing in the clinic and infusion area. In May of 2023, the clinic moved locations to a space intentionally designed for the SCD clinic including eight exam rooms (2 of which were outfitted to accommodate GYN exams) and an embedded infusion therapy unit with10 chairs. The staff for the SCD clinic also increased to include additional clinic nurses, infusion nurses, social workers, and medical assistants. By the end of 2024, our interventions lead to a three-fold increase in affiliated patients with a clinic population that included 810 adults living with SCD. Changes in the population, clinic space and staffing are noted in Table 2.

**Table 2:** SCD center cohort population, clinic space and staffing

	2019	2023	2024
Patient population (center database)	273 persons	621 persons	810 persons
Clinic Space	6 exam rooms 3 infusion chairs	8 exam rooms 10 infusion chairs	8 exam rooms 10 infusion chairs
Clinic Staff	1 scheduler 1 medical assistant 2 clinic RN 2 infusion RN 1 RN care coordinator 1 social worker 2 advance practice providers 1 FTE SCD specialist 0.5 FTE PCP	1 scheduler 2 medical assistants 3 clinic RN 4 infusion RN 3 social workers 1 pharmacist 1 transition coordinator 3 advance practice providers 1.25 FTE SCD specialist 0.5 FTE PCP	2 schedulers 3 medical assistants 4 clinic RN 4 infusion RN 1 transition coordinator 3 social workers 1 pharmacist 3 advance practice providers 1.25 FTE SCD specialist 0.5 FTE PCP

### Routine clinic visits

For optimal care delivery, individuals with SCD should be seen at least annually in the SCD center based on recommendations published by the NIH expert panel and the National Alliance of Sickle Cell Centers. (13,14) Many affected individuals require more frequent visits based on their individual complications, medications and treatment. The total number of unique patients seen annually for routine (non-acute) clinic visits are shown in Table 3 below for each year of study demonstrates the three-fold increase in capacity to provide non-acute care visits. By 2024, approximately 88.5% of our unique patients (717/810) living with SCD were seen in the outpatient clinic in 2024 for routine, non-acute care.

**Table 3:** Routine clinic visits, infusion therapy use, and acute care utilization pre and post implementation of a comprehensive SCD center

Year	Implementation Stage	Unique Patients in SCD Center Database	Unique Patients Seen in Clinic Annually	Total Routine Clinic Visits (per year)	Infusion Therapy Unit (ITU) Visits	ED Treat & Discharge Visits	ED → Inpatient Admissions	Inpatient-Only Admissions
2017	Pre-implementation	273	UNK	964	177	999	344	130
2018	Pre-implementation	273	UNK	1,040	140	1,101	331	103
2022	Post-implementation	621	547	1,629	160	927	288	147
2023	Post-implementation	753	675	4,659	1,275	831	246	164
2024	Post-implementation	810	717	4,875	2,782	837	262	183

### Infusion therapy unit visits

The infusion therapy unit (ITU) also called a day hospital in some settings was established to improve the delivery of pain management, decrease acute care use and to provide for blood transfusions and crizanlizumab (a SCD-modifying therapy) infusions. Prior to 2019, there was not a dedicated infusion area (for persons with SCD) resulting in few patients who were able to receive acute pain management outside of the ED. The ITU was open starting in Feb 2019 from 8am to 6pm daily during the week and was only available to patients with SCD within the Center Cohort. This intervention to improve access for acute outpatient pain management contributed to a 15-fold increase in the ITU visits. Data shown reflect mostly ITU visits for acute pain management (approximately 75% of the daily volume is for acute pain crisis management).

### Acute care utilization

**Fig. 3:**
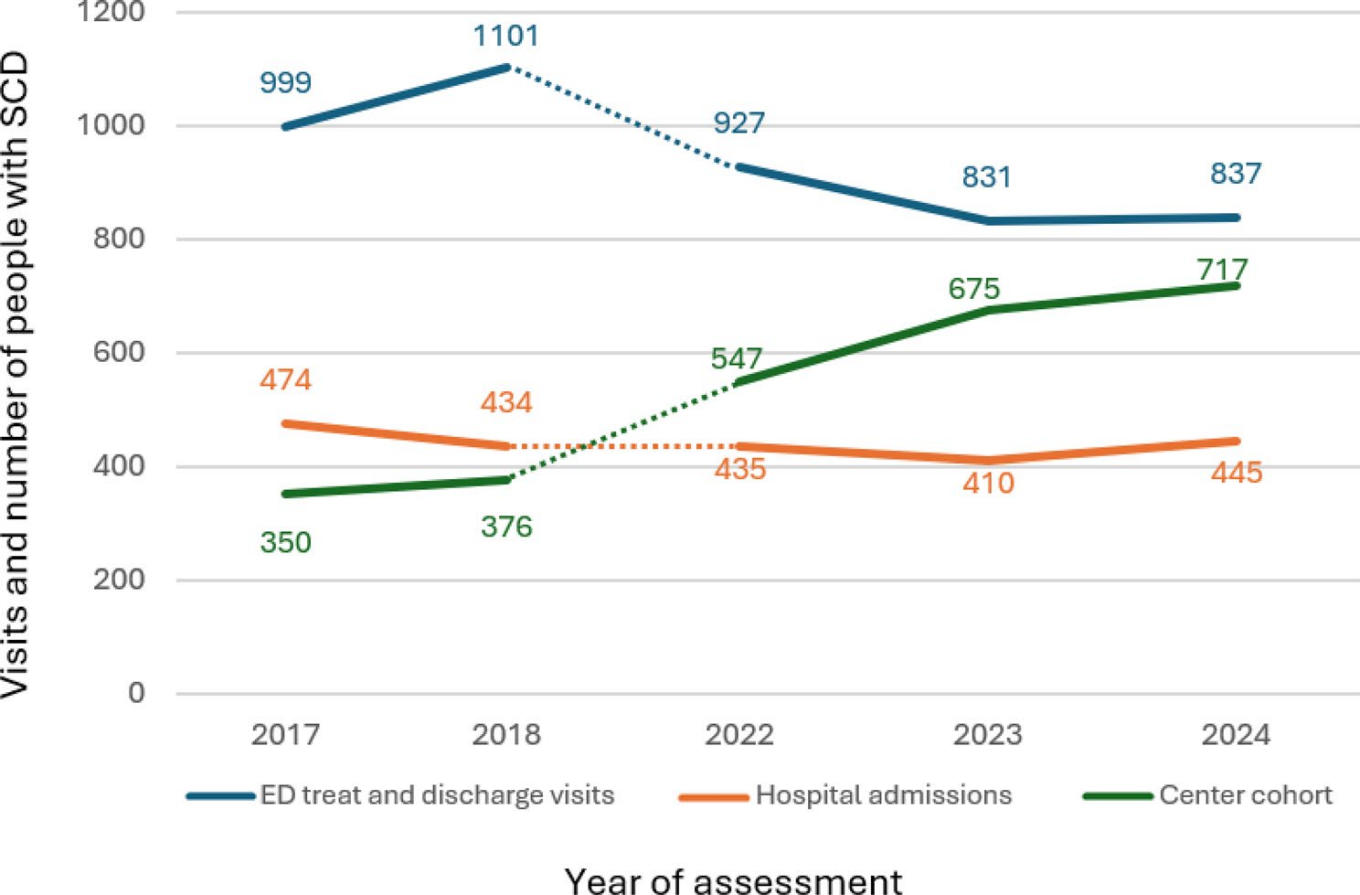
Graphic representation of comparison between ED visits and hospital admissions from 2017–2018 to 2022–2024 for the UAB SCD Population (total) and those identified in the Center Cohort (Green)

To assess the value proposition of the adult SCD center for the entire population of people with SCD seeking care at UAB, additional assessments of acute care use were undertaken. Data shown in Table 3; Fig. 3 demonstrate the associate decline in ED and inpatient visits to UAB hospitals. Hospitalization data does not include persons admitted for transformative therapy (allogeneic stem cell transplant or gene therapy) or patients that did not originate from the UAB health system (transferred for higher level of care) as these admissions do not relate to the provision of local care. While the number of persons presenting to the ED decreased, the proportion of patients admitted from the ED remained stable (23–26%). The admission rate from the ITU has remained < 10% (data not shown). The inpatient only admissions in Table 3 include patients admitted from the ITU but also include patients admitted for post-operative observation/management and for obstetric indications. Importantly some people with SCD still had acute pain during non-clinic hours and required urgent or ED care. However, if the acute pain crisis was uncomplicated (without fever, shortness of breath or neurologic symptoms), they often chose to wait for the ITU to open in order to receive expedited, more specialized attention.

Due to the substantial growth of the SCD Center Cohort from 2017 to 2024, it is necessary to also evaluate acute care visits per unique individual. This is especially important due to leadership concerns that the increased SCD population would lead to an increase in overall acute care use. These results are shown in Table 4 below based on both the total UAB SCD population and by those in the UAB Center Cohort. Acute visits per person were consistently lower for the cohort of patients in the UAB Center Cohort compared to the general population.

**Table 4:** Acute care utilization patterns among adults with SCD at UAB pre and post implementation of a comprehensive SCD center

	Acute Visits per Patient per year		Acute visits for UAB SCD Population						Acute visits for SCD Center Cohort					
Year	UAB (all)	SCD Center patients	Total patients	0	1	2–10	11–20	> 20	Total patients	0	1	2–10	11–20	> 20
2017	2.621	2.320	562	33.5%	28.6%	33.5%	2.3%	2.1%	350	53.4%	16.3%	25.1%	2.9%	2.3%
2018	2.597	2.157	591	34.9%	27.6%	32.5%	3.2%	1.9%	376	54.8%	18.4%	21.5%	3.2%	2.1%
2022	1.764	1.338	772	44.2%	25.8%	27.1%	2.2%	0.8%	547	62.3%	15.5%	19.9%	1.6%	0.5%
2023	1.420	1.084	874	50.0%	25.6%	21.9%	2.1%	0.5%	675	64.1%	18.1%	15.4%	2.1%	0.3%
2024	1.457	1.205	880	52.2%	24.2%	21.3%	1.7%	0.7%	717	63.7%	17.3%	17.0%	1.3%	0.7%

### Persons with SCD with frequent acute care use

Table 4 above shows the total number of patients with SCD seen at UAB during the period from 2017 to 2024 and those patients specifically followed in the UAB Center Cohort. This table highlights both the large number of persons who did not require acute care at all as well as the reduction in the number patients with high acute care use (> 10 acute visits/year) especially in the Center Cohort. There are a small proportion of people with SCD who have very frequent acute pain events that result in a very high frequency of ED and hospital visits. It is important to ensure this population is receiving optimal SCD treatment, evaluation and treatment for co-morbid psychiatric conditions and outpatient pain management. Starting in 2019, patients with very frequent use (> 10 acute care visits/year) were reviewed weekly in multi-disciplinary (MTD) meetings (in addition to other patients with significant complications) to tailor their care. Our MTD meetings included physicians, advance practice providers, social work, nurses, case managers, medical assistants, primary care provider, psychologist and (when needed) blood bank specialists. From 2019 to 2023, patients with very high acute care or those with other special needs (neurocognitive complications of SCD or other concerns) were assigned to a nurse care coordinator who checked-in with them weekly to assess any acute needs. This nurse coordinator was covered by the UAB institution through an internal program until the end of 2023. During 2023, we also identified patients (some in this group) who had symptoms of opioid-induced hyperalgesia to offer them a change in management to buprenorphine-naloxone for outpatient care. This program is highlighted in a separate publication [23]. It is also important to see that many people with SCD did not require any ED or hospitalization visits.

In the general analysis as well as in the sub analysis, the proportion of individuals with SCD with zero ED/hospital visits increases annually ranging from 33.5% in 2017 to 52.5% in 2024 among the general cohort and for persons followed within the SCD center, that figure ranges from 53.4% in 2017 to 63.7% in 2024. The reasons for the increase in the number of persons in the SCD clinic who did not require acute care are multifactorial but likely include increased use of disease modifying therapy, access to the ITU (to by pass the ED), availability of home pain medications, more patients with SCD with less acute care needs in the clinical program, etc.). The main unifying theme is that access to a SCD specialist/center lead to an increase in the number of persons who did not often require acute care. A further analysis of this population is in progress to better understand the primary driver(s) for these improvements.

### Persons in the UAB SCD population who were seen in emergency department for acute care but not followed by the SCD center

As in every hospital system, there are persons seen in emergency setting who do not receive their routine care at that same institution. Prior to 2019, there were no efforts in place to identify these people living with SCD and link them to comprehensive care or recommend they follow-up within their “home” institution to improve continuity of care. After the initiation of the SCD center, collaboration efforts with the ED staff and a data-driven intervention tool (alert) within the EMR allowed the team to identify these individuals and approach them for integration in the SCD Center. At times, people not followed at UAB declined the invitation to be seen in the SCD Center and preferred to maintain their care with another provider or were not local to the area (visiting). However, many individuals opted to move their care to UAB. Importantly, everyone living with SCD is also encouraged to seek emergency care at the same hospital where they are seen for routine care to improved care coordination whenever possible. Thus, patients seeking ONLY emergency/acute care at UAB who had the ability to obtain care locally with their own provider, were also encouraged to seek care at their “home” hospital. This practice is done to improve quality of care and ensure that people receiving acute pain management can be followed by the team and providers most familiar with their care.

In parallel with the significant decrease in acute visits/person/year at UAB post-implementation, we found a corresponding (significant) increase in the number of routine, non-acute outpatient clinic visits per person per year. Overall, mean acute visits per person per year decreased from 2.11 to 1.26, with the change for those independent between timeframes of 2.43 to 1.06 (p-value < 0.001) and the change for those who are present in both periods of 1.77 to 1.43 (p-value = 0.0108). The mean number of routine clinic visits per person per year increased with the implementation of the comprehensive SCD center from 2.65 to 5.32, with the change for those independent between timeframes of 2.77 to 4.81 (p-value < 0.001), and the change for those who are present in both periods of 2.60 to 6.27 (p-value < 0.001). Results shown in Table 5.

**Table 5:** Data here are only from SCD Center Cohort patients. Data include the number of acute care visits/patient/year and the number of routine clinic visits/patient/year. Included below are (A) All patients and (B) Patients who were seen either pre or post-implementation and (C) Patients who were seen in the SCD Center before and after implementation

		Mean (median) visits per person per year before SCD center (2017–2018)	Mean (median) visits per person per year after SCD center (2022–2024)	p-value
Acute visits	All patients included	2.11 (0.50)	1.26 (0.33)	N/A
	Clinic patients in only one period (not in both)	2.43 (0.50)	1.06 (0)	**< 0.001** (Nonparametric Wilcoxon rank sum test)
	People represented in both periods*	1.77 (0.50)	1.43 (0.33)	**0.148** (Nonparametric signed rank test)
Routine outpatient visits	All patients included	2.65 (2)	5.32 (4)	N/A
	People represented in only one period	2.77 (2)	4.81 (3.67)	**< 0.001** (Nonparametric Wilcoxon rank sum test)
	People represented in both periods	2.60 (2.5)	6.27 (4.33)	**< 0.001** (Nonparametric sign test)

^*^There are three outliers with absolute differences between time periods > 28 visits per year excluded for symmetry assumption of signed rank test

## Discussion

In this study, we demonstrate that the planned implementation of a NASCC-aligned comprehensive SCD center was associated with meaningful improvements in care delivery and utilization outcomes. Specifically, we observed a significant increase in the number of individuals who wanted to be seen in the SCD center with an improved adherence to scheduled outpatient clinic visits. The increase in internal referrals to the SCD center (from the ED, hospital and other clinics) resulted in a reduction in acute care utilization, including ED visits and hospitalizations. Together, these findings support the value of comprehensive, multidisciplinary SCD centers as a structural intervention to improve both engagement in care and health system utilization for people living with SCD.

Access to comprehensive outpatient preventative care is a necessity for people with chronic disease to improve outcomes. Data has demonstrated that regular longitudinal care is strongly associated with optimized disease-modifying therapy use, preventive screening, and early identification and treatment of complications [5]. The observed increase in outpatient (clinic) visits/patient seen at UAB likely reflects multiple center-level factors, including care coordination, standardized follow-up processes, and the development of trusted patient–provider relationships within a dedicated SCD care environment. Prior studies have shown that fragmented care and inconsistent access to SCD specialists have contributed to poor engagement in outpatient care; [24] our findings suggest that centralized, disease-specific, dedicated care can mitigate these barriers.

Our systematic approach to implementation of a NASCC-aligned comprehensive SCD center resulted in an increase in referrals via multiple pathways. The first was the initiation of the formalized transition program (from pediatric to adult care) to prevent loss to follow-up. The second major effort was undertaken as part of an NIH-funded project starting in 2023 (Recruitment and Engagement in Care to Impact Practice Enhancement), NCT06385886.) to enhance the identification and recruitment of unaffiliated persons with SCD into specialized care. This project involves the use of a dedicated linkage coordinator and identified pathways to improve the referral process including ensuring community clinicians had increased awareness of the SCD center. The use of these care pathways will be discussed in future publications to highlight the role of comprehensive centers both as direct care providers, and as hubs within the broader system of care.

Our adoption of a plan for horizontal service integration ensured that people with SCD received individualized care plans throughout the hospital system. This practice increases the delivery of guideline-based care and ensures uniformity at the system level. To be successful, horizontal care requires a supportive hospital administration and step-wise implementation. The use of ICP in people with SCD is a guideline-based intervention to enhance personalized care delivery [25, 26]. The plans are discussed during patients’ annual comprehensive visit to ensure the affected individual (person living with SCD) agrees with the ICP and it is placed in the EMR in a centralized location. The use of ICP also helps other physicians less comfortable with SCD management feel comfortable with designated treatment plans and enhances inter-professional collaboration.

Our results demonstrated reductions in acute care utilization despite a significantly increased patient population. This finding is both clinically and economically meaningful. Emergency department visits and hospitalizations for acute pain crisis and other SCD-related complications are costly, disruptive to patients’ lives, and often reflect gaps in outpatient management. The observed decrease in acute care results from the combined implementation of an infusion therapy unit, individualized care plans, increase in SCD-modifying therapies, increased preventive care, and proactive symptom management all of which require a robust multi-disciplinary team. Overall, these findings align with prior evidence that structured outpatient SCD programs including the use of day hospitals/infusion units are associated with fewer acute care encounters and improved patient-reported outcomes [24]. 

Importantly, these improvements occurred in a population historically affected by significant structural barriers, including insurance instability, transportation challenges, and stigma within health care settings [27–29]. The success of the SCD center model highlights the importance of care delivery interventions that address both clinical needs and social determinants of health. Dedicated staff roles especially social workers and nurse care coordinators are particularly critical to achieve sustainable improvements in care delivery.

This study has several limitations. Although the data collection methods were established prospectively, the analysis was performed retrospectively. Utilization data presented here do not include care received outside of the UAB health system. Additionally, the analysis does not include a patient-level assessment of ED use converted to ITU management (i.e. the extent to which ED episodes per patient were converted into acute infusion episodes per patient). The data shown from the ITU visits includes patients receiving crizanlizumab and blood transfusions as well as patients receiving acute pain management because the same codes are used in all instances. However, internal data reports show that the ITU is used for acute pain management for 75% of visits (not included here). Further, evaluation of the use of SCD-modifying therapies including chronic transfusion, hydroxyurea, crizanlizumab and novel therapies as well as changes in pain management also contributed to the findings but require additional discussion. Longer follow-up is needed to assess the impacts of improved outpatient clinic care on organ damage, mortality, and patient-reported quality of life. Nevertheless, the consistency of findings across multiple utilization and engagement metrics strengthens the overall finding that a multi-disciplinary, comprehensive SCD center is associated with meaningful improvements in care delivery.

In conclusion, our findings provide real-world evidence that comprehensive SCD centers can improve outpatient engagement, expand access to care for affected individuals, and reduce acute care. These results have important implications for health systems, payers, and policymakers, particularly as national efforts increasingly emphasize value-based care and equity-focused models for chronic disease management. These data highlight the importance of ensuring persons with SCD are referred to a comprehensive SCD center and that centers are appropriately staffed to ensure optimal patient engagement. This should be considered a foundational strategy to improve outcomes for individuals living with SCD.

## Data Availability

The data that support the findings of this study are available from the corresponding author upon reasonable request.
